# Changes in the Oral-Health-Related Quality of Life of Thai patients with oral lichen planus after topical corticosteroid treatment: a 1-month longitudinal study

**DOI:** 10.1186/s12903-023-03603-w

**Published:** 2023-11-21

**Authors:** Witchapat Kengtong, Pornpan Piboonratanakit, Sudaduang Krisdapong

**Affiliations:** 1 Maharat Nakhon Ratchasima Hospital, Nakhon Ratchasima, 30000 Thailand; 2 Department of Oral Medicine, Chulalongkorn University, Bangkok, 10330 Thailand; 3 Research Unit in Oral Diseases, Chulalongkorn University, Bangkok, 10330 Thailand; 4 Dental Public Health Researcher (Freelance), Bangkok, Thailand

**Keywords:** Oral lichen planus, Quality of life, Oral-health-related quality of life, Oral impact on daily performances index, OIDP, Topical corticosteroid treatment

## Abstract

**Background:**

Oral lichen planus (OLP) is a chronic inflammatory disease of the oral cavity that affects many patients’ daily living activities. Topical corticosteroids are the first-line drug for treating OLP. The Oral Impact on Daily Performances index (OIDP) is an Oral-Health-Related Quality of Life (OHRQoL) measure developed to assess the ultimate impacts. The aims of this study were to evaluate the clinical, pain and OHRQoL responses after treating OLP patients with topical corticosteroids for 1 month, and secondly to assess the relationships of changes in the clinical sign score, pain score, and OHRQoL.

**Methods:**

Seventy-two OLP patients were treated by topical corticosteroids based on their dentists’ clinical judgments. Clinical and patient-based outcomes were assessed at baseline and follow-up visit. The clinical outcomes were evaluated by the highest and total Thongprasom sign score. Patient-based outcomes were evaluated by numeric rating scale (NRS) and OIDP. The self-rated overall changes in quality of life during the 1-month treatment period using the Patient Global Impression of Change (PGIC) were also recorded at the follow-up visit.

**Results:**

This study comprised 59 women and 13 men. All clinical and patient-based outcomes were significantly reduced after 1-month treatment with topical corticosteroids (*P* < 0.01). The most commonly affected activities were Emotional stability, followed by Eating and Cleaning the oral cavity. Forty-six percent reported the same or up to moderately improved, while 54% had a greatly improved quality of life as assessed by PGIC. There were no significant differences in the improvement of clinical and patient-based outcomes between these groups. There were significant relationships between the differences in the highest Thongprasom sign score and the differences in total Thongprasom sign score (r = 0.293; *P* < 0.05), and the differences in total OIDP percentage score and the difference in pain score (r = 0.427; *P* < 0.001). The differences in the total Thongprasom sign score also significantly related to the difference in the total OIDP percentage score (r = 0.335; *P* < 0.01).

**Conclusions:**

Topical corticosteroids were significantly effective after 1-month treatment of OLP based on the clinical and patient-based outcomes. The OHRQoL improvement was significantly related to the reductions in pain and clinical severity.

**Trial registration:**

The trial was registered at the Thai Clinical Trials Registry (TCTR identifier: TCTR 20221110001).

## Background

Oral lichen planus (OLP) is a common chronic inflammatory disease of the oral cavity that affects the daily living activities in many patients. Most OLP patients are middle-aged women; the most common lesion sites are the buccal mucosa followed by the tongue, and gingiva [1]. OLP occurs in several forms, with the classic forms being white lesions presenting as reticular, papular, or plaque-like patterns and red lesions presenting as atrophic and ulcerative areas [2]. The main symptoms of OLP are a burning sensation when eating hot and spicy foods or severe chronic pain in the oral cavity [3]. The goal of treating OLP is to relieve symptomatic pain and reduce inflammation. The first-line drugs for treating OLP are corticosteroids taken in topical forms that can be used in various types and preparations, such as fluocinolone acetonide oral paste and dexamethasone mouthwash [4–6]. The advantages of topical corticosteroids are the lower risk of systemic steroid effects, such as hypertension, gastric ulcers, bone mineral loss, or adrenal suppression, however, they have an increased risk of oral candidiasis when used over a prolonged period [7].

Currently, OLP clinical trials should use a tripartite approach comprising clinical signs, symptoms, and quality of life [8]. Chainani-Wu et al. [9] recommended using an instrument that was sensitive and easy to apply when evaluating the signs of OLP. For clinical evaluation, several OLP studies in many countries including Thailand have applied the Thongprasom sign scoring system [5, 10–12]. This scoring system takes the size and clinical severity of each OLP lesion into account. However, the weak point of the Thongprasom sign scoring system is that it does not take into account the number of lesions. Only the highest score is assigned for a patient regardless of the number of lesions.

Because OLP was a chronic inflammatory disease that is very difficult to completely cure, patients suffer from a burning sensation, pain, and discomfort [13]. Pain rating scales, e.g. numeric rating scale (NRS) and visual analog scale (VAS), are widely accepted and are more useful for assessing OLP symptoms [9]. Several OLP longitudinal studies used these scales for assessing OLP symptoms [11, 14–16]. Moreover, the concept of patient-based outcome measures has been used to determine impaired oral health from the patient’s perspective using the Oral-Health-Related Quality of Life (OHRQoL) [17]. These perspectives can be measured by several indices, such as the Oral Health Impact Profile (OHIP), Oral Health-Related Quality of Life (OHQoL) and Chronic Oral Mucosal Disease Questionnaire (COMDQ) [18]. Several OLP longitudinal studies chose OHIP or OHQoL as instruments to determine the patient-centered outcomes along with the efficacy of the drugs after treatment [12, 15, 16]. Furthermore, COMDQ was one of the measurements used in a phase II study of clobetasol patches for treating OLP [14].

The Oral Impact on Daily Performances Index (OIDP) [19] is another OHRQoL measure developed to assess the ultimate impacts, i.e. difficulties in daily life performances that are the consequences of intermediate impacts, e.g. pain, discomfort, functional limitations, and dissatisfaction with appearance, thus, avoiding repetition in measuring the impacts. If pain as the intermediate impact leads to difficulty in eating as the ultimate impact, OIDP will consider only difficulty in eating. In oral medicine, OIDP was used to assess the oral impacts attributed to angular cheilitis, and geographic tongue in the Portuguese version [20]. The Arabic OIDP version was applied to assess the relationship between OHRQoL and oral lesions related to skin lesions [21]. In Thailand, this index has been validated in all age groups and widely applied in several dental specialties’ clinical research and in Thailand national health surveys [22–25]. There was also an investigation of OHRQoL attributed to recurrent aphthous stomatitis in Thai children in a national oral health survey using OIDP [26]. Yiemstan et al. [27] also used the Thai OIDP version for investigating the associations of OHRQoL and pain with clinical signs based on the Thongprasom sign scoring system in OLP patients and found the significant associations of clinical severity with the intensity of oral impacts as well as pain.

The previous studies about oral mucosal lesions that used OIDP were cross-sectional and case-control studies, whereas most OLP longitudinal studies used OHIP and OHQoL for OHRQoL evaluation. None of the OLP longitudinal studies used OIDP to measure OHRQoL. Therefore, the aim of this study was to evaluate the clinical, pain and OHRQoL responses after a 1-month treatment with topical corticosteroid in OLP patients. Secondly, to assess the relationships of the changes in the clinical sign score, pain score, and OHRQoL.

## Methods

### Study subjects

The sample size was calculated using 80% power and 95% confidence level according to data from McGrath et al. [15]. They assessed the OHRQoL in patients with OLP treated with topical betamethasone using OHQoL-UK and OHIP-14 questionnaires. The effect size (ρ) was 0.31, thus, the estimated total sample size was 66. To compensate for error or loss of participants during follow up, the sample size was increased by 10%, resulting in 72 patients.

The patients were recruited from the Oral Medicine clinic, Faculty of Dentistry, Chulalongkorn University. The inclusion criteria were being over 18 years old, patients diagnosed with OLP or compatible with OLP following clinical and histopathological criteria [28], oral lichenoid drug reaction (OLDR) patients with a history of taking a related medication, patients receiving topical corticosteroid for OLP treatment, follow-up patients requiring follow-up appointment for 1 month, and patients with written informed consent. The exclusion criteria were pregnant patients, patients with a history of smoking within six months, patients who were treated with other modalities or had other types of oral mucosal lesions, or oral lichenoid contact lesion patients. The interviewer was trained and calibrated with an expert in using the OIDP index. The inter-rater agreement was good with an intraclass correlation coefficient (ICC) of 0.877.

### Data collection

#### Demographic data

Age, sex, systemic condition, medication taking and OLP duration since the first diagnosis were collected from the history taking at the first visit. In addition, the OLP locations (e.g. lips, labial mucosa, buccal mucosa, gingiva, tongue, floor of the mouth, and palate), experience with OLP treatment (i.e., new or follow-up patient) and prescribed topical corticosteroids based on their dentists’ clinical judgments were also recorded.

#### Clinical outcomes

Regarding the clinical data collection, the highest Thongprasom sign score was based on the original Thongprasom sign scoring system, i.e., “0” indicated no lesions; “1” indicated white striae only, “2” indicated white striae with an atrophic area of less than 1 cm^2^, “3” indicated white striae with an atrophic area of 1 cm^2^ or more, “4” indicated white striae with an erosive area of less than 1 cm^2^ and “5” indicated white striae with an erosive area of 1 cm^2^ or more (5). The highest Thongprasom sign score obtained from the most severe lesion ranging from 0 to 5, was assigned to the patient.

As mentioned earlier that Thongprasom sign scoring system did not take into account the number of OLP lesions, so we purposed the total Thongprasom sign score, which was the sum of all lesions’ Thongprasom sign scores. The OLP distribution was recorded as the outer and inner lips, right and left buccal mucosa, upper right, upper central, upper left, lower left, lower central, and lower right gingiva, dorsal, left and right ventrolateral tongue, floor of the mouth, hard palate, and soft palate [9]. Because the possible maximum number of OLP oral lesions was 16, the total Thongprasom sign scores ranged from 0 to 80. The highest and total Thongprasom sign scores were recorded at baseline and the follow-up visit.

#### Patient-based outcomes

The patients were asked about the chief symptoms of OLP that made them visit the dentist at baseline, such as a burning sensation, ulceration, and roughness. The patients could have more than 1 symptom or have no symptoms. In the aspect of pain, the OLP patients reported their NRS score for their worst pain during 1 month at baseline and the follow-up visit [29], ranging from 0 to 10: “0” meant no pain at all, and “10” meant the worst pain imaginable.

The patients’ OHRQoL was assessed using the Thai version of the OIDP [22]. The patients were asked about the OLP that limited their eight daily activities at baseline and the 1-month follow-up visit. The eight activities consisted of Eating, Speaking, Cleaning the oral cavity, Relaxing including sleeping, Smiling, laughing without embarrassment, Emotional stability, Carrying out major work and Social contact. The frequency and severity scores of difficulties, if any, on performing each activity were recorded. Due to the chronic nature of OLP, frequency scores on a regular basis was used: “0” meant never affected, “1” meant once a month, “2” meant twice a month, “3” meant once or twice a week “4” meant three to four times a week and “5” meant every or almost every day. The severity scores were scored as: “0” meant never affected daily life, “1” meant very low impact, “2” meant low impact, “3” meant moderate impact, “4” meant high impact, and “5” meant very high impact. The frequency and severity scores of difficulties on performing each activity were multiplied, resulting in a performance score ranging from 0 to 25. The sum of the eight performance scores ranging from 0 to 200 was divided by 2 to get a total OIDP percentage score ranging from 0 to 100. The higher scores indicated a poorer OHRQoL [19].

**Fig. 1:**
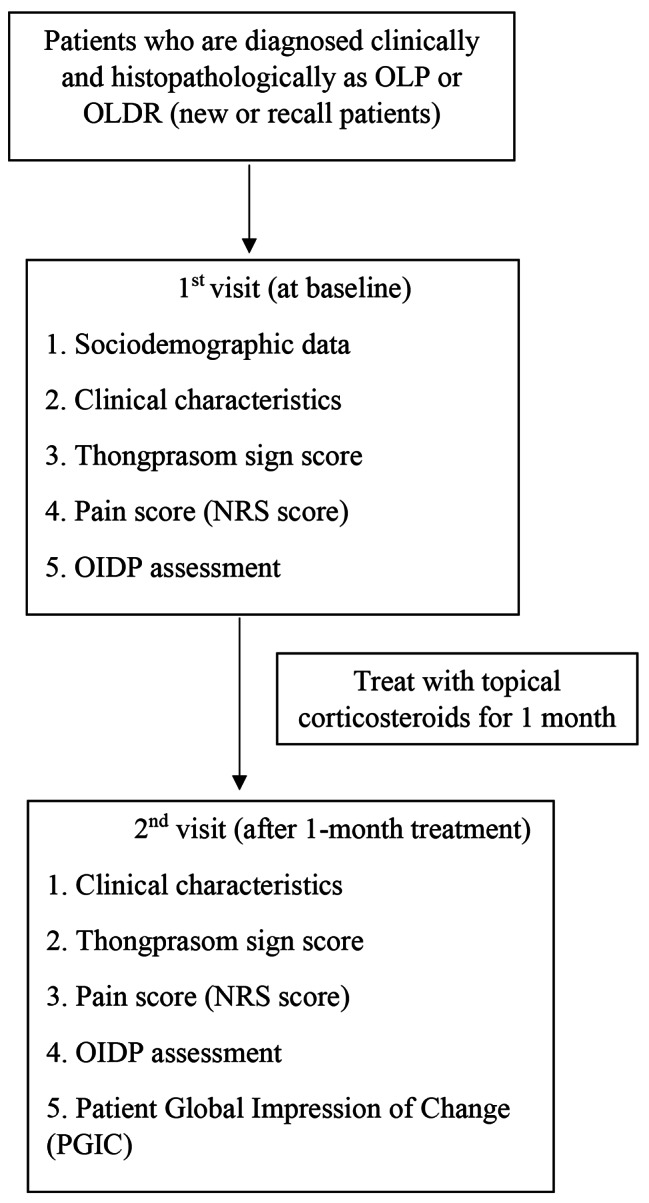
Patient flow chart (Participant flow)

At the 1-month follow-up visit, the differences in each outcome were calculated by subtracting the follow-up data from the baseline data. Negative values represented a worsened effect, whereas positive values represented an improved effect. The differences in the average values were determined. In addition, the OLP patients were asked to self-rate the overall changes in quality of life during the 1-month treatment period. The rating criteria from the Patient Global Impression of Change (PGIC) measure [30] were used. The PGIC was categorized on a 7-point scale as “severely worsened”, “moderately worsened”, “minimally worsened”, “no change”, “minimally improved”, “moderately improved”, and “greatly improved”. The patient flow chart is shown in Fig. 1.

### Data analysis

Statistical calculations were performed with SPSS software (SPSS 22.0 for Windows; SPSS, Chicago, IL, USA). Descriptive statistics were used for the demographic and clinical data. The Kolmogorov-Smirnov normality tests were performed to determine the normal data distribution in each outcome. The pain score and total Thongprasom sign score between baseline and after the 1-month treatment were assessed using the paired t-test. The highest Thongprasom sign score, total OIDP percentage score, and performance scores of each daily activity between baseline and after the 1-month treatment were analyzed by the Wilcoxon signed-rank test. The differences in the highest Thongprasom sign score, total Thongprasom sign score, pain score and total OIDP percentage score between two groups of patients who reported no changes to moderately-improved and greatly-improved PGIC were tested using the Mann-Whitney U test. Pearson’s correlation was used to assess the relationship between the differences in the total OIDP percentage score, highest Thongprasom sign score, total Thongprasom sign score and pain score. The significance level was set at *P* < 0.05.

## Results

### OLP patient characteristics

The 72 patients (100% response rate) in this study consisted of 59 women (81.9%) and 13 men (18.1%). The mean age of the patients in this study was 53.3 ± 12.4 years old (range 22–81 years old). Forty-six patients (63.9%) were between 30 and 60 years old. Twenty-three patients (31.9%) were more than 60 years old and 3 patients (4.2%) were less than 30 years old. Fifty-one patients (70.8%) had their OLP less than 1 year before seeing the dentists, however, 14 patients (19.4%) were diagnosed 1–3 years earlier. Seven patients (9.8%) had their lesions for more than 3 years. The mean duration of OLP was 21.3 ± 30.1 months (range 1–120 months).

Regarding patients’ systemic conditions, 40 patients (55.6%) reported no medical problem and were not taking any medication. Twenty-two patients (30.6%) were diagnosed as dyslipidemia and took anti-dyslipidemia drugs (e.g. simvastatin, rosuvastatin, etc.) Furthermore, 20 (27.8%) of the patients had hypertension and 9 (12.5%) had diabetes mellitus. Both diseases required more than one group of drugs, namely, 27 patients (37.5%) required hypertensive drugs (e.g. amlodipine, atenolol, enalapril etc.) and 13 patients (18.1%) required diabetic drugs (e.g. metformin, glipizide, etc.). Four patients (5.6%) with thyroid disease and 2 patients (2.8%) with gout had taken levothyroxine and allopurinol, respectively.

Some patients reported more than one chief complaint at the first visit. Common chief complaints were burning sensation (80.6%), ulceration (65.3%), and rough oral mucosal surface (19.4%). One patient had no symptom (1.4%). Sixty patients (83.3%) were new patients and 12 patients (16.7%) were follow-up patients.

OLP occurred in various sites in the oral cavity, with the most commonly affected area being the buccal mucosa (80.6%) followed by gingiva (76.4%), tongue (18.1%), lips (16.7%), and floor of the mouth (11.1%). Less than 10% of the patients demonstrated OLP at the palate (6.9%) and labial mucosa (5.6%).

The most frequently prescribed topical corticosteroids were 0.1% fluocinolone acetonide in orabase (37.5%), followed by 0.05% dexamethasone mouthwash (31.9%), 0.1% triamcinolone mouthwash (16.6%) and 0.05% fluocinolone mouthwash (15.3%). Other topical corticosteroids, 0.05% clobetasol propionate in orabase (2.8%) and 0.1% fluocinolone solution (1.4%), were infrequently prescribed. (Table 1)

**Table 1:** Baseline characteristics and clinical data of the OLP patients (N = 72)

Characteristics	N (%)
**Sex**	
- Women - Men	59 (81.9) 13 (18.1)
**Age**	
Mean ± SD: 53.3 ± 12.4 years	
Range: 22–81 years	
- Less than 30 years	3 (4.2)
- 30–60 years	46 (63.9)
- More than 60 years	23 (31.9)
**Duration**	
Mean ± SD: 21.3 ± 30.1 months	
Range: 1–120 months	
- Less than one year	51 (70.8)
- 1–3 years	14 (19.4)
- More than three years	7 (9.8)
**Systemic condition**	
- None - Dyslipidemia - Hypertension - Diabetes mellitus - Thyroid disease - Gout	40 (55.6) 22 (30.6) 20 (27.8) 9 (12.5) 4 (5.6) 2 (2.8)
**Medication taking**	
- None - Anti-dyslipidemia drugs - Anti-hypertensive drugs - Anti-diabetic drugs - Levothyroxine - Allopurinol	40 (55.6) 22 (30.6) 27 (37.5) 13 (18.1) 4 (5.6) 2 (2.8)
**Chief complaint**	
- Burning sensation - Ulceration - Roughness - No symptoms	58 (80.6) 47 (65.3) 14 (19.4) 1 (1.4)
**Experienced about OLP treatment**	
- New patients - Follow-up patients	60 (83.3) 12 (16.7)
**Locations**	
- Lips - Labial mucosa - Buccal mucosa - Gingiva - Tongue - Floor of mouth - Palate	12 (16.7) 4 (5.6) 58 (80.6) 55 (76.4) 13 (18.1) 8 (11.1) 5 (6.9)
**Topical corticosteroids treated**	
- 0.1% Fluocinolone acetonide in orabase - 0.05% Clobetasol propionate in orabase - 0.05% Dexamethasone mouthwash - 0.1% Triamcinolone mouthwash - 0.05% Fluocinolone mouthwash - 0.1% Fluocinolone solution	27 (37.5) 2 (2.8) 23 (31.9) 12 (16.6) 11 (15.3) 1 (1.4)

### Clinical outcomes

At baseline, the median highest Thongprasom sign score was 3.0 ± 1.0 (range 2–5) and the mean total Thongprasom sign score was 13.7 ± 6.8 (range 2–30). At the 1-month follow-up visit, the median highest Thongprasom sign score was 2.0 ± 0.0 (range 1–4), thus, the difference in scores from baseline was 1.0 ± 1.0 (range − 1 to 3). The mean total Thongprasom sign score was 8.7 ± 5.2 (range 0–26), thus, the difference from baseline was 5.2 ± 4.1 (range − 1 to 16). Both clinical outcomes significantly improved after 1-month treatment with topical corticosteroids (*P* < 0.01). (Table 2)

**Table 2:** Comparison of the highest Thongprasom sign score, total Thongprasom sign score, pain score, and total OIDP percentage score outcomes between baseline and 1-month follow- up visit (N = 72)

Index	Average (range)		
	Baseline	Follow-up	Differences^a^
Highest Thongprasom sign score	3.0 ± 1.0 (2–5)	2.0 ± 0.0^c^ (1–4)	1.0 ± 1.0 (-1–3)
Total Thongprasom sign score	13.7 ± 6.8 (2–30)	8.7 ± 5.2^b^ (0–26)	5.2 ± 4.1 (-1–16)
Pain score	6.7 ± 2.6 (0–10)	2.9 ± 2.2^b^ (0–10)	3.8 ± 2.5 (0–10)
Total OIDP percentage score	17.3 ± 13.3 (1.5–48)	2.0 ± 4.5^c^ (0–31)	14.5 ± 12.6 (1.5–47)

^a^ Subtracted the follow-up data from baseline data and sharing with each n. Negative values represented a worsened effect, whereas positive values represented an improved effect

^b^ Mean ± SD, statistically significant as compared with baseline at *P* < 0.01 (paired t-test)

^c^ Median ± IQR, statistically significant as compared with baseline at *P* < 0.01 (Wilcoxon signed-rank test)

### Patient-based outcomes

At baseline, the mean pain score was 6.7 ± 2.6 (range 0–10) and the median total OIDP percentage score was 17.3 ± 13.3 (range 1.5–48). At the 1-month follow-up visit, the mean pain score was 2.9 ± 2.2 (range 0–10) and the reduced pain score was 3.8 ± 2.5 (range 0–10). The pain score significantly reduced after 1-month treatment (*P* < 0.01). The median total OIDP percentage score after 1-month treatment was 2.0 ± 4.5 (range 0–31). Difference in total OIDP percentage score was 14.5 ± 12.6 (range 1.5–47). The reduction from baseline was statistically significant (*P* < 0.01). (Table 2)

The three most commonly affected performances were Emotional stability (94.4%), Eating (86.1%) and Cleaning the oral cavity (65.3%). Fewer patients had problems in Social contact (20.8%), Carrying out major work (13.9%), Speaking (12.5%) and Smiling, laughing without embarrassment (12.5%). No patient reported relaxing disturbance due to OLP. At baseline, the performance with the highest performance score was Eating (15.0 ± 10.0), followed by Emotional stability (12.0 ± 11.5), Cleaning the oral cavity (12.0 ± 11.0), and Smiling, laughing without embarrassment (12.0 ± 10.5). The median performance scores of the other three activities, i.e. Speaking, Social contact, and Carrying out major work and were less than 10 (6.0 ± 12.5, 4.0 ± 6.0, 5.0 ± 4.5, respectively). At the follow-up visit, the median performance scores of Eating (3.5 ± 6.0), Emotional stability (1.0 ± 4.0), and Cleaning the oral cavity (0.0 ± 1.0) were significantly decreased compared with those at baseline (*P* < 0.01). In addition, the OIDP performance scores of the other four activities; Speaking, Smiling, laughing without embarrassment, Social contact, and Carrying out major work, were also significantly decreased compared with those at baseline (*P* < 0.05). (Table 3)

**Table 3:** Comparison of each performances’ outcomes between baseline and 1-month follow-up visit (N = 72)

Performance	N (%)	Performance score Median ± IQR (range)		
		Baseline	Follow-up	Differences^a^
Eating	62 (86.1)	15.0 ± 10.0 (1–25)	3.5 ± 6.0^c^ (0–20)	10.0 ± 9.3 (-1–25)
Speaking	9 (12.5)	6.0 ± 12.5 (0–20)	0.0 ± 2.0^b^ (0–8)	6.0 ± 12.5 (-8–16)
Cleaning	47 (65.3)	12.0 ± 11.0 (1–20)	0.0 ± 1.0^c^ (0–16)	12.0 ± 9.0 (1–20)
Relaxing	0	0	0	0
Smiling	9 (12.5)	12.0 ± 10.5 (1–20)	0.0 ± 2.0^b^ (0–9)	11.0 ± 11.0 (1–20)
Emotional stability	68 (94.4)	12.0 ± 11.5 (2–25)	1.0 ± 4.0^c^ (0–20)	9.0 ± 10.8 (-2–25)
Working	10 (13.9)	5.0 ± 4.5 (2–16)	1.0 ± 2.5^b^ (0–9)	3.0 ± 2.8 (0–8)
Social contact	15 (20.8)	4.0 ± 6.0 (2–25)	0.0 ± 1.0^b^ (0–6)	4.0 ± 7.0 (0–25)

^a^ Subtracted the follow-up data from baseline data and sharing with each n. Negative values represented a worsened effect, whereas positive values represented an improved effect

^b^ Statistically significant as compared with baseline at *P* < 0.05 (Wilcoxon signed-rank test)

^c^ Statistically significant as compared with baseline at *P* < 0.01 (Wilcoxon signed-rank test)

### Patient global impression of change (PGIC)

After 1-month treatment with topical corticosteroid, none of the patients reported severely worsened, moderately worsened or minimally worsened quality of life. One patient (1.4%) reported no changes after treatment. Four patients (5.6%) described themselves as minimally improved, 28 patients (38.9%) felt moderately improved, and 39 patients (54.2%) felt greatly improved. Therefore, we made 2 groups of patients that were the same or up to moderately improved (45.8%) and greatly improved (54.2%) quality of life groups to compare the reduced clinical and patient-based outcomes. The differences in total OIDP percentage score were 12.5 ± 8.5 in the same or up to moderately improved group and 15.0 ± 15.0 in the greatly improved group (*P* = 0.079). In contrast, the differences of other outcomes, consisting of the highest Thongprasom sign score, total Thongprasom sign score, and pain scores demonstrated similar values or slight differences between the groups (*P* = 0.528, *P* = 0.820, and *P* = 0.233, respectively). (Table 4)

**Table 4:** Comparison of the differences in clinical and patient-based outcomes between groups of patients who reported up to moderately improved and greatly improved (N = 72)

Differences	Differences^a^ Median ± IQR (range)		*P* value^c^
	Up to moderately improved ^b^ (N = 33)	Greatly improved (N = 39)	
Highest Thongprasom sign score	1.0 ± 0.5 (0–3)	1.0 ± 1.0 (0–3)	0.528
Total Thongprasom sign score	4.0 ± 6.0 (0–12)	4.0 ± 7.0 (-1–16)	0.820
Pain score	4.0 ± 3.5 (1–10)	4.0 ± 4.0 (1–10)	0.233
Total OIDP percentage score	12.5 ± 8.5 (1.5–31.5)	15.0 ± 15.0 (4.5–47)	0.079

^a^ Subtracted the follow-up data from baseline data and sharing with each n. Negative values represented a worsened effect, whereas positive values represented an improved effect

^b^ Including 1 no changes, 4 minimally improved and 28 moderately improved patients

^c^ Statistically analyzed with Mann-Whitney U test

### Relationships between the differences in the clinical and patient-based outcomes

The difference in total OIDP percentage score and difference in pain score demonstrated a significant relationship (r = 0.427; *P* < 0.001) and the difference in total OIDP percentage score and difference in total Thongprasom sign score was significantly related (r = 0.335; *P* < 0.01). There was a significant relationship between the difference in the highest Thongprasom sign score and the difference in total Thongprasom sign score (r = 0.293; *P* = 0.012). No significant relationship was found between the difference in the highest Thongprasom sign score and the difference in total OIDP percentage score (r = 0.198; *P* = 0.096), or between the differences in the highest Thongprasom sign score or total Thongprasom score and the difference in pain score (r = 0.109; *P* = 0.362 and r = 0.118; *P* = 0.324, respectively). (Table 5)

**Table 5:** Relationships of the differences among the total OIDP percentage score, highest Thongprasom sign score, total Thongprasom sign score and pain score

Differences	Differences					
	Total Thongprasom score		Pain score		Total OIDP percentage score	
	r	*P* value	r	*P* value	r	*P* value
Highest Thongprasom sign score	0.293	0.012^a^	0.109	0.362	0.198	0.096
Total Thongprasom sign score			0.118	0.324	0.335	0.004^b^
Pain score					0.427	< 0.001^c^

^a^ Statistically significant at *P* < 0.05 (Pearson’s correlation)

^b^ Statistically significant at *P* < 0.01 (Pearson’s correlation)

^c^ Statistically significant at *P* < 0.001 (Pearson’s correlation)

## Discussion

Nowadays, the importance of utilizing patient-based outcomes alongside clinical outcomes has been recognized. The current study’s findings could support patient-centered care in evidence-based dentistry in relation to OLP treatment with topical corticosteroid. Most of the patients participating in this study benefited from using topical corticosteroids, as demonstrated by the improvement in the clinical and patient-based outcomes. We found that the highest and total Thongprasom sign scores for OLP were significantly decreased after 1-month treatment with topical corticosteroids compared with baseline. Several reviews revealed that topical corticosteroids are clinically effective and are considered the treatment of choice for OLP [31, 32]. Thongprasom et al. [5] found no significant clinical improvement in 2 weeks, however, after 4 weeks, more than 60% of OLP patients who used 0.1% fluocinolone acetonide in orabase had the highest Thongprasom sign score of less than 2. Furthermore, there have been multiple clinical trials supporting that improvement in clinical sign scores and OLP symptoms can be detected after at least 4 weeks [10, 33]. Hegarty et al. [16] found that topical corticosteroids reduced the signs and symptoms of OLP within 3–4 weeks, thus, these drugs were effective in the early treatment of symptomatic OLP.

OLP affects patient’s quality of life, however, effective treatment can improve their ability to perform everyday activities [16]. One of the goals of OLP treatment with topical corticosteroids is to have the patients recover their ability to perform the basic activities of daily life, such as eating, drinking, or tooth brushing [34]. In the present study, the three activities with the highest prevalence were Emotional stability followed by Eating, and Cleaning the oral cavity. The total OIDP percentage score was also significantly decreased. Moreover, all performances, except for Relaxing including sleeping, in which no patient reported difficulty before receiving treatment, improved by more than 80% after 1 month. These results indicate that the OLP patients achieved a better quality of life in every important domain after 1-month treatment with topical corticosteroids. Our results corresponded to those of a previous study in which OLP patients had a significant negative impact on OHIP (e.g. functional limitation, physical disability, physical pain and psychological discomfort) and all these impacts were improved after topical corticosteroid treatment [12]. Previous longitudinal OLP studies using OHIP and OHQoL as OHRQoL assessment tools also found a significant improvement in OHRQoL in OLP patients after treating them with topical corticosteroids by 3–6 weeks [15, 16]. Hambly et al. [6] reported that their patients could consume spicy foods or felt more confident after using topical corticosteroids to treat their OLP.

The PGIC, the patients’ perception on their changing OHRQoL, results revealed that most patients reported improvement in various scales. However, the difference in the reduced clinical, pain and OIDP scores between those reporting up to moderately improved and greatly improved were not significant. Feine et al. [35] demonstrated that patients’ reports of relief following treatment were often inaccurate. Errors in remembering symptoms increased over longer durations. These results corresponded with those in Santonocito et al. [11] where more than half of the OLP patients felt well, even though the erosive areas had not changed much. Thus, patient’s perception on relief or change after treatment might be less reliable due to the distortions in their memory, compared with measuring OHRQoL at two time points and calculating the change in the score. However, it should be noticed that the difference in total OIDP percentage score between the up to moderately improved and greatly improved quality of life groups was marked (12.5 vs. 15.0), while those of the other 3 parameters (highest Thongprasom sign score, total Thongprasom sign score and pain score) were similar. The reason for this is that evaluating quality of life by different instruments should obtain similar results, which agreed with a report that changes in OHIP scores correlated with changes in OHQoL scores after topical corticosteroid treatment [15].

When considering the relationships between the changes in the clinical and patient-based outcomes after 1-month treatment with topical corticosteroids, we found significant relationships for the differences in patient-based outcomes (pain score and total OIDP percentage score) and the differences in clinical outcomes (highest Thongprasom sign score and total Thongprasom sign scores). These findings were as expected and confirmed the abovementioned discussion on the close relationship between outcomes assessed by different instruments belonging to the same concepts, either clinical or patient-based. Regarding the relationships between the difference in clinical outcomes and the difference in patient-based outcomes, the total Thongprasom sign score better correlated with patient-based outcomes, compared with the highest Thongprasom sign score. A possible explanation might relate to the different impacts from different OLP locations. A study reported that OLP occurring at different locations caused different degrees of symptoms, minor symptoms could be expected for lesions occurring at the buccal/labial mucosa, gingiva and palate, while the symptoms could be greater for tongue lesions [9]. In our study, many patients experienced multiple lesions because the sum of the location percentages was much higher than 100. The assessment of the patient-based outcomes was for a person whose perception was derived from all the existing lesions, while lesions located at different locations cause different impacts. Therefore, the total Thongprasom sign score conceptually corresponds to the patient-based outcome assessment. This might explain the better relationship of the total Thongprasom sign score with the patient-based outcomes than the highest Thongprasom sign score as found in our study.

Our study’s weak point was the various types and forms of topical corticosteroids with different potencies used in the clinic. Most of our patients received 0.1% fluocinolone acetonide in orabase and 0.05% dexamethasone mouthwash for treating OLP. Thongprasom et al. [5] compared the efficacy of 0.1% fluocinolone acetonide in orabase versus 0.1% triamcinolone acetonide in orabase and found that the clinical OLP improvement in the 0.1% fluocinolone acetonide in orabase treatment group was better than the 0.1% triamcinolone acetonide in orabase treatment group after 4 weeks. Several clinical trials demonstrated that the OLP treatment response from topical corticosteroids in various types and forms relieved pain or burning sensation and reduced the extent and severity of the OLP [10, 11]. Buajeeb et al. [10] found that 0.1% fluocinolone acetonide gel was safe and effective for treating OLP. Santonocito et al. [11] found that 0.05% clobetasol oral gel was more effective in treating OLP than anti-inflammatory mouthwash. Although the clinical and patient-based outcomes from using these topical corticosteroids demonstrated improvements, many previous clinical trials found that each type and concentration of each corticosteroid can result in different clinical outcomes [5, 6, 10, 14, 16]. These factors may impact the relationship between the clinical outcomes and patient-based outcomes.

Another weak point was the different characteristics of the patients in this study, such as age, sex, duration of OLP, systemic conditions, medication, experience in OLP treatment and types of topical corticosteroid. Moreover, different numbers and locations of OLP in the oral cavity may have impacted the patient-based results and relationship between the clinical outcomes and patient-based outcomes as discussed above. The current study’s objectives did not include the analyses of the association between these factors and patients’ OHRQoL. Therefore, further studies using a larger sample size are recommended to explore the impacts of such factors, for example, type of topical corticosteroid, on clinical and patient-based outcomes after treatment. Such findings may be helpful for medical communication or further insurance planning. Lastly, the current study used the OIDP index, while many other studies have utilized other OHRQoL indices such as the OHIP. Thus, findings from different indices cannot be compared, which consequently, limit the building up knowledge in this field. Future research using the same index as studies in other countries is recommended in order to compare and combine the results through more impactful analyses, which would bring a more understanding on the impacts of OLP on dental patients globally and to support the pragmatic use of OHRQoL index in dental clinics.

## Conclusion

After a 1-month OLP treatment with topical corticosteroids, the highest Thongprasom sign score, total Thongprasom sign score, and pain score were significantly reduced. The patients’ overall OHRQoL and daily performances were significantly improved, except for Relaxing including sleeping of which none of the patients reported a problem before treatment. The differences in the clinical and patient-based outcomes in the group reporting up to moderately improved did not significantly differ from those in the group reporting a greatly improved quality of life. There were significant relationships between the improvement in two clinical outcomes and the improvement in two patient-based outcomes. The decrease in the total Thongprasom sign score was also significantly related to the improvement in OHRQoL.

## Data Availability

All data generated or analyzed during this study are included in this published article.

## Abbreviations

OLP: Oral lichen planus
NRS: Numeric rating scale
VAS: Visual analog scale
OHRQoL: Oral-Health-Related Quality of Life
OHIP: Oral Health Impact Profile
OHQoL: Oral Health-Related Quality of Life
COMDQ: Chronic Oral Mucosal Disease Questionnaire
OIDP: Oral Impact on Daily Performances Index
OLDR: Oral lichenoid drug reaction
ICC: Intraclass correlation coefficient
PGIC: Patient Global Impression of Change
